# The relative importance of households as a source of variation in child malnutrition: a multilevel analysis in India

**DOI:** 10.1186/s12939-021-01563-7

**Published:** 2021-10-12

**Authors:** Anoop Jain, Justin Rodgers, Rockli Kim, S. V. Subramanian

**Affiliations:** 1 Global Health & Social Medicine, Harvard Medical School, Boston, MA 02115 USA; 2 Harvard Center for Population and Development Studies, Cambridge, MA 02138 USA; 3 Division of Health Policy & Management, College of Health Science, Korea University, Seoul, 02841 South Korea; 4 Interdisciplinary Program in Precision Public Health, Department of Public Health Sciences, Graduate School of Korea University, Seoul, 02841 South Korea; 5 Harvard Center for Population and Development Studies, MA 02138 Cambridge, USA; 6 Department of Social and Behavioral Sciences, Harvard T.H. Chan School of Public Health, Boston, MA 02115 USA

**Keywords:** Social determinants, Child malnutrition, India, Multilevel modeling, Population health, Omitted level, Households

## Abstract

**Background:**

Child malnutrition remains a major public health issue in India. Along with myriad upstream and social determinants of these adverse outcomes, recent studies have highlighted regional differences in mean child malnutrition rates. This research helps policy makers look between urban and rural communities and states to take a population-level approach to addressing the root causes of child malnutrition. However, one gap in this between-population approach has been the omission of households as a unit of analysis. Households could represent important sources of variation in child malnutrition within communities, districts, and states.

**Methods:**

Using the fourth round of India’s National Family Health Survey from 2015 to 2016, we analyzed four and five-level multilevel models to estimate the proportion of variation in child malnutrition attributable to states, districts, communities, households, and children.

**Results:**

Overall, we found that of the four levels that children were nested in (households, communities, districts, and states), the greatest proportion of variation in child height-for-age Z score, weight-for-age Z score, weight-for-height Z score, hemoglobin, birthweight, stunting, underweight, wasting, anemia, and low birthweight was attributable to households. Furthermore, we found that when the household level is omitted from models, the variance estimates for communities and children are overestimated.

**Conclusions:**

These findings highlight the importance of households as an important source of clustering and variation in child malnutrition outcomes. As such, policies and interventions should address household-level social determinants, such as asset and social deprivations, in order to prevent poor child growth outcomes among the most vulnerable households in India.

**Supplementary Information:**

The online version contains supplementary material available at 10.1186/s12939-021-01563-7.

## Background

Child malnutrition remains a major public health issue in India. Often indicated by measures of child anthropometry and low hemoglobin levels, over 30% of the world’s stunted children (i.e. children with < − 2 SD height-for-age Z score) lived in India in 2017 [1]. Furthermore, the prevalence of child wasting (< − 2 SD weight-for-height Z score), underweight (< − 2 SD weight-for-age Z score), anemia (< 11.0 g/deciliter), and low birthweight (< 2500 g) was 15.7, 32.7, 59.7, and 21.4%, respectively [1, 2]. In addition to being associated with an increased risk of infectious disease [3], child malnutrition is linked with impaired cognitive development that can lead to poor long-term educational and economic outcomes [4]. Furthermore, mild to moderate child malnutrition is associated with increased child mortality [2].

Social determinants such as household socioeconomic status, intergenerational poverty, and inadequate environmental conditions are all associated with child malnutrition [5–7]. Additionally, some studies highlight regional differences in mean child malnutrition rates. For example, districts with high rates of stunting are clustered in north and central India [8]. Other studies have shown that states such as Tamil Nadu are high performing in terms of child malnutrition outcomes, while other states, such as Bihar, Uttar Pradesh, Odisha, Madhya Pradesh, and Gujarat perform quite poorly [9]. Furthermore, multilevel analyses reveal that variations in malnutrition are most attributable to states and urban and rural communities as opposed to districts [10, 11]. Corruption, variations in health policy and service delivery, and differences in health care spending are some of the reasons associated with disparate child malnutrition outcomes across states and communities in India [11–13].

However, one gap in these multilevel analyses and population approaches has been the omission of households as a unit of analysis. This omission may stem in part from the assumption that within-population distributions remain constant over time and across populations [14, 15]. As a result, the environments, such as households, that people are embedded in within communities, districts, or states are often disregarded. However, a strictly population-level approach does not account for heterogeneity at lower levels. This is demonstrated by the fact that over 93% of the variation in child height-for-age Z score (HAZ) in India was attributable to between-individual variations [11]. Similarly, another study showed that 80–85% of the variation in child hemoglobin and anthropometry in India was due to within-population differences [14]. Households are important sites of etiologic action given previously established relationships between housing characteristics, conditions, and amenities, and health [15]. As such, households could represent an important unit of investigation in multilevel analyses within communities, districts, and states.

Given this background, the purpose of this paper is to examine the proportion of variation in child stunting, wasting, underweight, anemia, and low birthweight attributable to the household level relative to commonly analyzed levels such as communities, districts, and states. We conducted this study using data from India’s most recent National Family Health Survey (NFHS), and to our knowledge, no other papers using this dataset have included households as a unit of analysis. This research is significant given that India’s National Nutrition Strategy (NNS) seeks to dramatically reduce the burden of child malnutrition in the coming years [16]. Achieving this goal will require an approach that targets at-risk individuals and households given how much of the variation in child malnutrition is attributable to lower levels [17–20] in addition to a population-level strategy to reduce average rates of child malnutrition across states in India [11, 21]. Therefore, this research is important as it builds on prior studies by examining households, a lower level of inferential targeting, as a possible source of variation in child malnutrition. Understanding the role households play could help policy makers intervene both between and within populations in order to improve child malnutrition throughout India.

## Methods

### The National Family Health Survey 2015–2016

We used the fourth round of the NFHS from 2015 to 2016 to conduct this study. Households were defined as a group of individuals who normally live together and take their meals from a common kitchen. Overall, this survey used a stratified two-stage sampling frame (states, and urban/rural areas within states) to select households and participants [22]. More specifically, households were selected from primary sampling units, defined as groups of adjacent households, which were villages in rural areas and census enumeration blocks in urban areas. We collectively refer to these village and census enumeration blocks as communities in this paper. As such, this dataset contains data from each of India’s 36 states/union territories, all 640 districts, 28,522 out of over 650,000 communities, 601,509 households, and 699,686 women between the ages of 15–49. For the purposes of this paper, the survey included data on a total of 259,627 children from 180,227 households.

### Variables and sample sizes

**Fig. 1:**
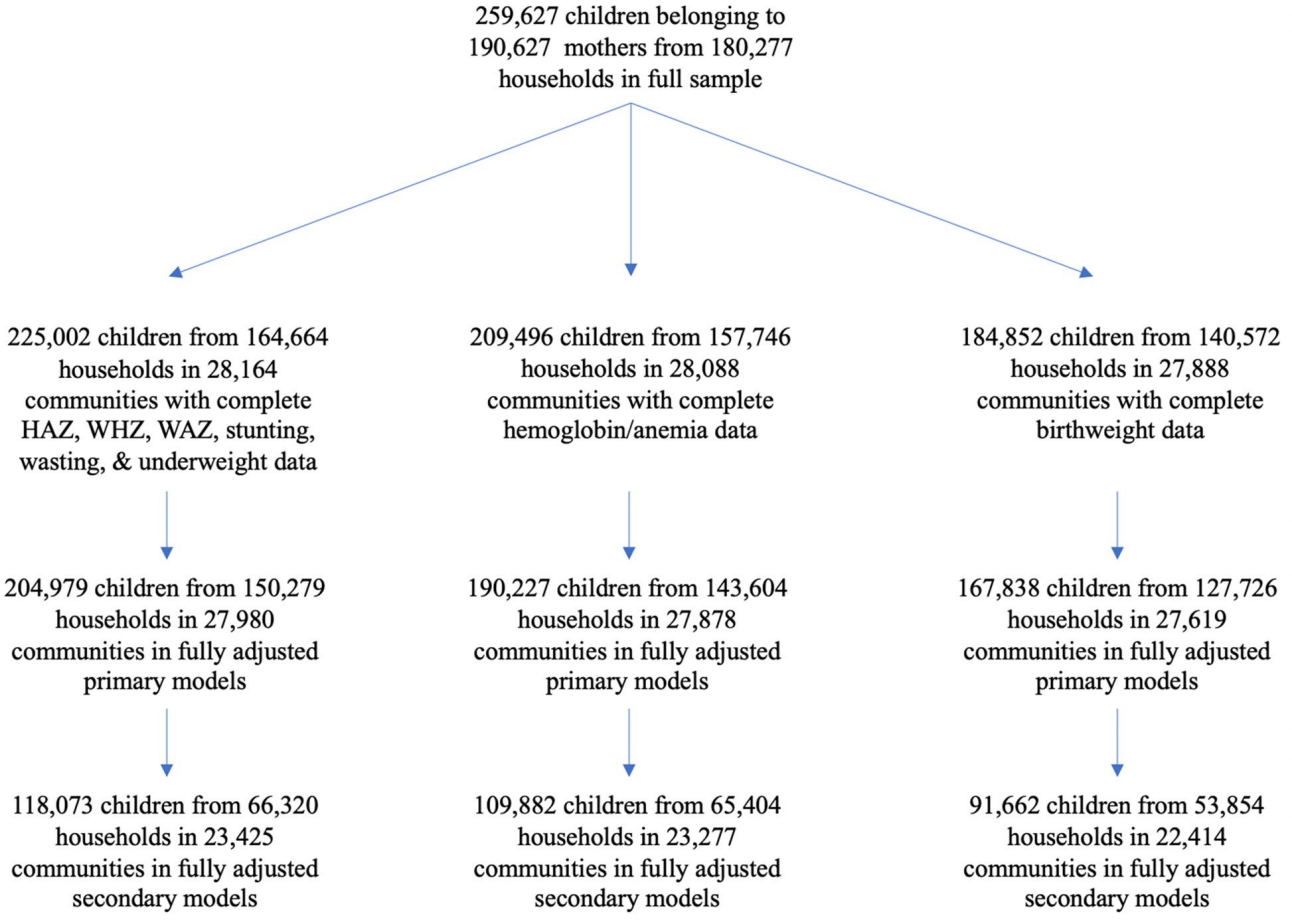
Flow chart of sample sizes for primary and secondary analyses for all outcomes

The outcomes included in our analysis were child height-for-age Z score (HAZ), weight-for-height Z score (WHZ), weight-for-age Z score (WAZ), standardized hemoglobin measures (HB), and birthweight (measured in grams). We also included stunting (<− 2 SD HAZ), wasting (<− 2 SD WHZ), underweight (<− 2 SD WAZ), anemia (< 11.0 g/dL), and low birthweight (< 2500 g). The dataset had complete HAZ, WAZ, and WHZ data for 225,002 children from 164,664 households. The dataset had complete HB data for 209,496 children from 157,746 households, and birthweight data for 184,852 children from 140,572 households. Sample sizes are fully described in Fig. 1 below. The number of states/union territories [23] and districts (640) stayed the same in all the analyses hence they are not reported in the figure.

Covariates included 19 risk factors known to be associated with child malnutrition [7, 24, 25]. We included three different nutrition variables, early breastfeeding initiation, vitamin A supplementation (only asked in reference to children between 6 and 59 months), and the use of iodized salt. Each of these three variables was dichotomized yes/no. We included four environmental variables, household access to improved sanitation, household use of clean cooking fuel, safe child stool disposal, and household access to improved drinking water. These variables were also dichotomized yes/no. A total of six health coverage variables were included. These were whether the child experienced an infectious disease in the past two weeks, complete child vaccinations, presence of skilled birth attendant at birth, provision of oral rehydration therapy after diarrhea, care seeking for pneumonia, and whether family planning needs were met. All of these variables were dichotomized yes/no. Household wealth, mother’s level of education, and child’s birth order were included as socioeconomic risk factors. Households were dichotomized by either being in the poorest wealth quintile, or in all other quintiles. Mother’s education was dichotomized based on whether the mother had received no education or at least primary education. We dichotomized birth order as before or after the 6th birth. Finally, we included three maternal characteristic risk factors which were mother’s age at marriage, maternal height, and maternal body mass index (BMI). Age at marriage was dichotomized as above or below 18. Mother’s height was dichotomized as above and below 145 cm, and mother’s BMI was dichotomized as above and below 18.5 kg/m^2^.

After performing listwise deletion for all missing observations for the above covariates, our fully adjusted sample sizes were 204,979 children with HAZ, WAZ, and WHZ outcome data, 190,227 children with HB outcome data, and 167,838 children with birthweight outcome data.

### Statistical analysis

Our statistical analysis consisted of a series of four- and five- level models in order to compare the addition of households as a level of analysis. Additionally, each set of models included an ‘unadjusted’ and ‘adjusted’ model. The unadjusted models contained only child age (in months) and sex, while the fully adjusted models contained all 19 risk factors outlined above. Further, our analysis was composed of primary and secondary analyses. The primary analysis included the full sample of households, with varying numbers of children per household, whereas the secondary analysis focused on a restricted subsample composed of only those households with more than one child. This approach was taken in order to examine whether household variance estimates were attenuated by between-child estimates in households with more than one child.

#### Continuous outcomes

We used multilevel modeling to decompose the proportion of variation in our continuous outcomes – HAZ, WHZ, WAZ, HB, and birthweight – attributable to children at level one, nested in urban/rural communities at level two, districts at level three, and states at level four. Multilevel modeling is a statistical methodology commonly used in the field of public health to elucidate the effects of both compositional and contextual factors on health [26–28]. For this four-level model, we estimated equation (1), which took the basic form *Y*_*ijkl*_ = *β*_0_ + *β*_1_*X*_*ijkl*_ + (*e*_0*ijkl*_ + *u*_0*jkl*_ + *v*_0*kl*_ + *f*_0*l*_) where *Y*_*ijkl*_ is one of the outcomes for child *i* nested in community *j,* district *k,* and state *l.* In this model, *X*_*ijkl*_ is a vector of covariates, and the random effects *e*_0*ijkl*_, *u*_0*jkl*_, *v*_0*kl*_, and *f*_0*l*_ are the residual differentials for children, communities, districts, and states, respectively. We then included households such that we decomposed the proportion of variation in the same continuous outcomes attributable to children at level one, nested in households at level two, communities at level three, districts at level four, and states at level five. For this five-level model, we estimated equation (2), which took the basic form *Y*_*ijklm*_ = *β*_0_ + *β*_1_*X*_*ijkl*._ + (*e*_0*iyjkl*_ + *h*_0*yjkl*_ + *u*_0*jkl*_ + *v*_0*kl*_ + *f*_0*l*_) where *Y*_*iyjkl*_ is one of the outcomes for child *i* nested in household *y*, community *j,* district *k,* and state *l.* In this model, *X*_*iyjkl*_ is a vector of covariates, and the random effects *e*_0*iyjkl*_, *h*_0*yjkl*_, *u*_0*jkl*_, *v*_0*kl*_, and *f*_0*l*_ are the residual differentials for children, households, communities, districts, and states, respectively. In both the four-level and five-level models, the residual differentials for children, households, communities, districts, and states are assumed to be normally distributed with a mean of 0 and a variance of ${\sigma}_{e0}^2$, ${\sigma}_{h0}^2$, ${\sigma}_{u0}^2$, ${\sigma}_{v0}^2$, and ${\sigma}_{f0}^2$, respectively. The variances in the four and five-level models are the parameters of interest and signify the between-child (${\sigma}_{e0}^2\Big)$, between-household (${\sigma}_{h0}^2$), between-community (${\sigma}_{u0}^2$), between-district (${\sigma}_{v0}^2\Big)$, and between-state (${\sigma}_{f0}^2$) variations in child *i* experiencing the outcome. Finally, we repeated the five-level analysis for a subsample of households with more than one child.

#### Binary outcomes

For the binary outcomes stunting, wasting, underweight, anemia, and low birthweight, we estimated four level models for the probability of a child *i,* in community *j*, in district *k,* in state *l* experiencing the outcome *Y*_*ijkl*_ = 1 as equation (3) *logit*(*π*_*ijkl*_) = *β*_0_ + *β*_1_*X*_*ijkl*_ + (*u*_0*jkl*_ + *v*_0*kl*_ + *f*_0*l*_), where *π*_*ijkl*_ is the log odds of the outcome in child *i*, *X*_*ijkl*_ is a vector of covariates, and the random effects are the residual differentials for communities (*u*_0*jkl*_), districts (*v*_0*kl*_), and states (*f*_0*l*_). We then added households in order to decompose the proportion of variation in the same binary outcomes attributable to children at level one, nested in households at level two, communities at level three, districts at level four, and states at level five. For this five-level model, we estimated the probability of child *i,* in household *y,* in community *j*, in district *k,* in state *l* experiencing the outcome *Y*_*iyjkl*_ = 1 as equation (4) *logit*(*π*_*iyjkl*_) = *β*_0_ + *β*_1_*X*_*iyjkl*_ + (*h*_*yjkl*_ + *u*_0*jkl*_ + *v*_0*kl*_ + *f*_0*l*_). In this case, the random effects *h*_*yjkl*_, *u*_0*jkl*_, *v*_0*kl*_, and *f*_0*l*_ are the residual differentials for households, communities, districts, and states, respectively. The same assumptions and parameter definitions used for equations (1) and (2) were applied to equations (3) and (4). However, the variance of the lowest levels cannot be estimated when considering binary outcomes. As such, the child-level random effect and variance is not shown. Again, we repeated the five-level analysis for households with more than one child.

We used the Monte Carlo Markov Chain (MCMC) method with a burn-in of 5000 cycles monitored over 50,000 chain iterations in MLwiN 3.05 software to conduct the analysis and produce the estimates for the continuous and binary outcomes in the four and five level models [29, 30].

## Results

### Sample characteristics

In the full sample of the 225,002 children with anthropometric data, the mean HAZ value was − 1.48 (std. dev. 1.68). The average WAZ and WHZ values were − 1.52 (std. dev. 1.22) and − 0.97 (std. dev. 1.39), respectively. Approximately 38.3% of the children were stunted, 20.4% experienced wasting, and 34.5% were underweight. The average HAZ, WAZ, and WHZ values among the 128,197 children in households with more than one child were − 1.59 (std. dev. 1.69), − 1.60 (std. dev. 1.21), and − 0.98 (std. dev. 1.37). In the restricted sample, 41.2% of children were stunted, 36.7% were underweight, and 20.4% experienced wasting.

Of the full sample of 209,496 children with hemoglobin data, the average HB value was 10.6 (std. dev. 1.54). Approximately 57.5% of the children in our sample were anemic. In the restricted sample, the mean HB value was 10.5 (std. dev. 1.55) among the 119,668 children with complete hemoglobin data, and approximately 58.1% of the children were anemic. Finally, the average birthweight was 2.82 kg among the 184,852 children with birthweight data. Approximately 17.7% of the children were classified as having low birthweight. In the restricted sample, the mean birthweight was 2.81 kg among the 99,667 children with birthweight data, and 17.9% were classified as having low birthweight.

### Household level variance for continuous outcomes

We estimated the household level variance for HAZ, WHZ, WAZ, HB, and birthweight. These results are presented in Table 1. Overall, we found that the unadjusted and adjusted household variance estimates for HAZ were 0.47 (0.01) and 0.41 (0.01), respectively. For WHZ, the unadjusted and adjusted household variance estimates were 0.32 (0.007) and 0.30 (0.008). For WAZ, the unadjusted and adjusted household variance estimates were 0.36 (0.005) and 0.31 (0.005), respectively. For hemoglobin, the unadjusted household variance estimate was 0.33 (0.008), while the adjusted estimate was 0.32 (0.008). Finally, the unadjusted and adjusted household variance estimates for birthweight were 0.086 (0.002) and 0.082 (0.002), respectively. Overall, we found that except for the child level, the largest proportion of variation was attributable to households for HAZ, WHZ, WAZ, HB, and birthweight.

**Table 1:**
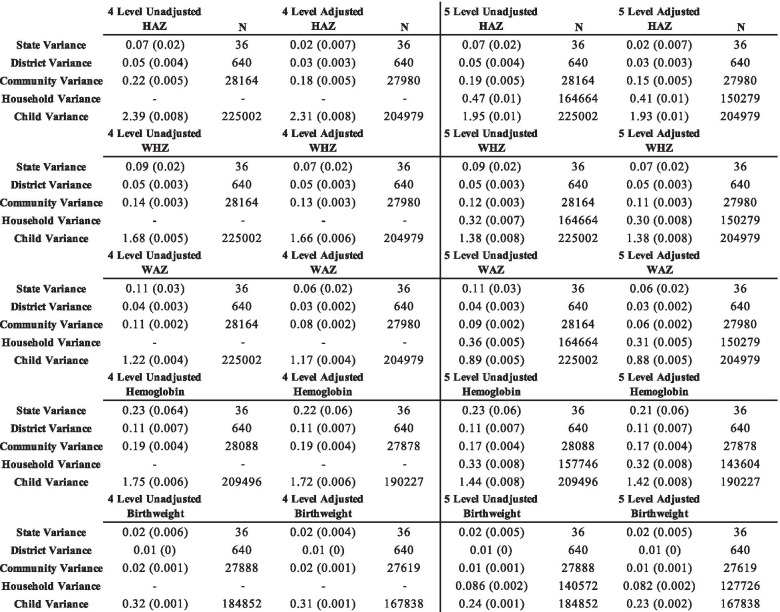
Four and five-level variance estimates (standard error) for continuous anthropometric, hemoglobin, and birthweight outcomes

### Household level variance for categorical outcomes

We estimated the household level variance for stunting, wasting, underweight, anemia, and low birthweight. These results are presented in Table 2. The unadjusted and adjusted household variance estimates for stunting were 0.27 (0.013) and 0.74 (0.09), respectively. For wasting, the unadjusted household variance estimate was 0.89 (0.04), and 0.87 (0.05) in the adjusted model. The unadjusted and adjusted household variance estimates for underweight were 1.29 (0.04) and 0.93 (0.35), respectively. The unadjusted and adjusted household variance estimates were both 0 (0) for anemia. Finally, for low birthweight, the unadjusted and adjusted household variance estimates were 1.49 (0.24) and 0.45 (0.48), respectively.

**Table 2:**
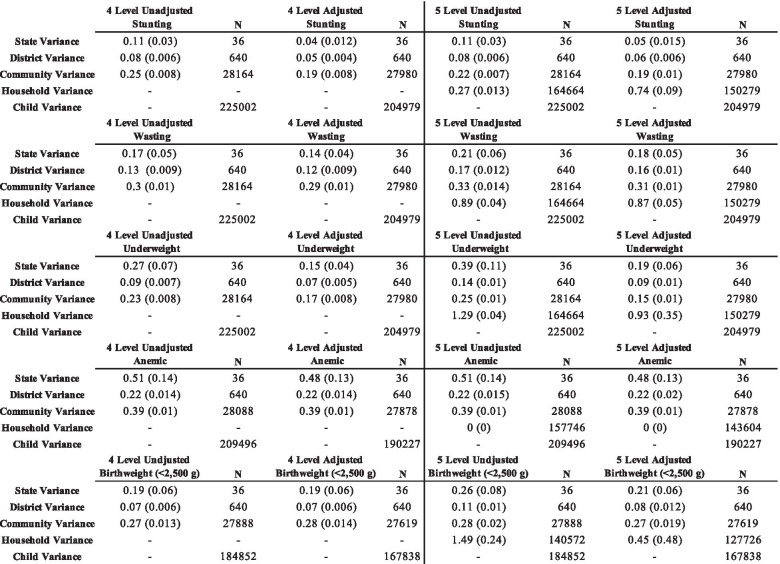
Four and five-level variance estimates (standard error) for stunting, wasting, underweight, anemia, and low birthweight

### Comparing four level and five level variance estimates

We found that state and district variance estimates remained stable between the four and five-level models for each of the continuous outcomes. However, we found differences in the community variance estimates between the fully adjusted four-level and fully adjusted five-level models for all five continuous outcomes. These differences are presented in Table 3. For HAZ, the four-level adjusted community-level variance estimate was 17% higher than the five-level adjusted community variance estimate. For WHZ, the four-level adjusted community-level variance estimate was 15% higher than the five-level adjusted community variance estimate. For WAZ, the four-level adjusted community-level variance estimate was 25% higher than the five-level adjusted community variance estimate. For HB, the four-level adjusted community-level variance estimate was 11% higher than the five-level adjusted community variance estimate. For birthweight, the four-level adjusted community-level variance estimate was 50% higher than the five-level adjusted community variance estimate.

**Table 3:**
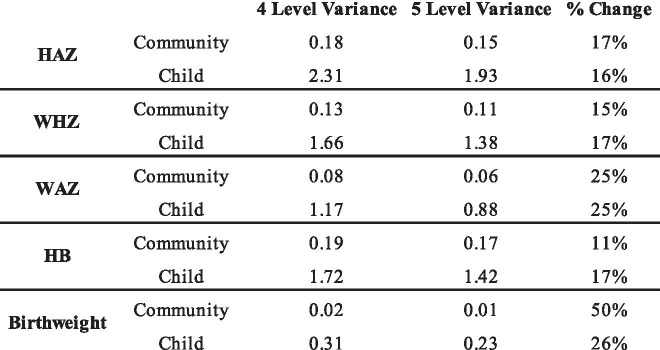
Differences between four and five level variance estimates in adjusted models for continuous outcomes

Similarly, we found that the adjusted four-level models overestimated the child-level variance for all five outcomes when compared to the adjusted five-level models. For HAZ, the four-level adjusted child-level variance estimate was 16% higher than the five-level adjusted community variance estimate. For WHZ, the four-level adjusted child-level variance estimate was17% higher than the five-level adjusted community variance estimate. For WAZ, the four-level adjusted child-level variance estimate was 25% higher than the five-level adjusted community variance estimate. For HB, the four-level adjusted child-level variance estimate was 17% higher than the five-level adjusted community variance estimate. Finally, for birthweight, the four-level adjusted child-level variance estimate was 26% higher than the five-level adjusted community variance estimate.

There were also differences in the variance estimates between the fully adjusted four-level and fully adjusted five-level models for the binary outcomes. For stunting, the state, district, and community variance estimates in the fully adjusted four-level model were 0.04 (0.012), 0.05 (0.004), and 0.19 (0.008), respectively, compared to 0.05 (0.015), 0.06 (0.006), and 0.19 (0.01) in the fully adjusted five-level model. For wasting, the state, district, and community variance estimates were 0.14 (0.04), 0.12 (0.009), and 0.29 (0.01), respectively, compared to 0.18 (0.05), 0.16 (0.01), and 0.31 (0.01) in the fully adjusted five-level model. For underweight, the state, district, and community variances in the fully adjusted four-level model were 0.15 (0.04), 0.07 (0.005), and 0.17 (0.008), respectively, compared to 0.19 (0.06), 0.09 (0.01), and 0.15 (0.01) in the fully adjusted five-level model. For low birthweight, the state, district, and community variances in the fully adjusted four-level model were 0.19 (0.06), 0.07 (0.006), and 0.28 (0.014), respectively, compared to 0.21 (0.06), 0.08 (0.012), and 0.27 (0.019) in the fully adjusted five-level model.

### Households with more than one child

We estimated the household level variance for all five continuous and binary outcomes in a subsample of the population containing homes with more than one child. These results are presented in Table 4. The unadjusted and adjusted household variance estimates for HAZ were 0.49 (0.01) and 0.42 (0.012), respectively. For WHZ, the unadjusted and adjusted household variance estimates were both 0.29 (0.008). For WAZ, the unadjusted and adjusted household variance estimates were 0.36 (0.006) and 0.31 (0.006), respectively. For HB, the unadjusted household variance estimate was 0.36 (0.006), while the adjusted estimate was 0.35 (0.01). The unadjusted and adjusted household variance estimates for birthweight were both 0.09 (0.002).

**Table 4:**
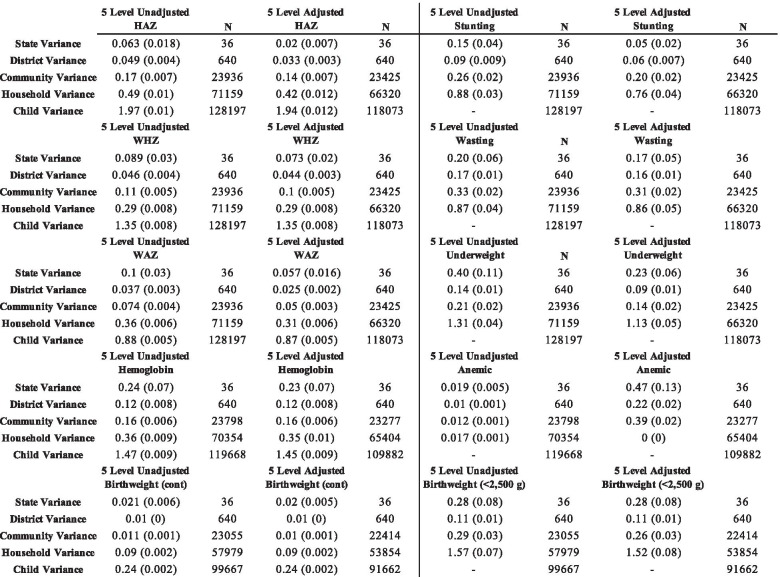
Five level variance estimates (standard error) for households with more than one child

For stunting, the unadjusted and adjusted household variance estimates were 0.88 (0.03) and 0.76 (0.04), respectively. The unadjusted and adjusted household variance estimates for wasting were 0.33 (0.02) and 0.31 (0.02). For underweight, the unadjusted household variance estimate was 1.31 (0.04) compared to 1.13 (0.05) in the adjusted model. For anemia, the unadjusted household variance estimate was 0.017 (0.001) while the adjusted variance was 0 (0). Finally, for birthweight, the unadjusted and adjusted household variance estimates were 1.57 (0.07) and 1.52 (0.08), respectively. Table 5 below shows a comparison between the household-level variance estimates between the full sample and households with more than one child.

**Table 5:**
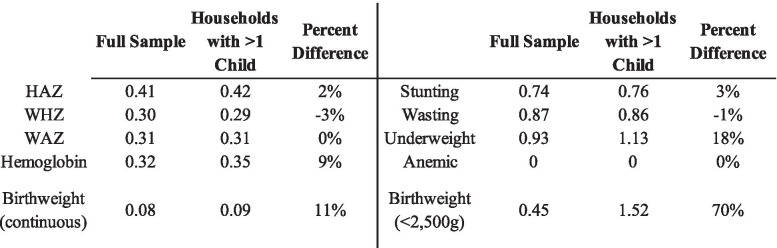
Comparison of household-level variance estimates between full sample and households with more than one child

## Discussion

This study had three salient findings. First, in the four-level models, the majority of the variance was attributable to the community and child levels. However, in the five-level models, of the four levels that children were nested in (households, communities, districts, and states), the greatest proportion of variation in child HAZ, WAZ, WHZ, HB, and birthweight was attributable to households. This was also the case for stunting, wasting, underweight, and low birthweight. It is possible this was not the case for anemia given the high prevalence of anemia in the overall sample, and because over 50% of the households with more than one child either had all children anemic or none anemic (described in supplementary Table 1). Second, we found that community and child-level variance estimates are overestimated in four-level models that omit the household level for HAZ, WAZ, WHZ, HB, and birthweight. Third, while the greatest proportion of the variation was still attributable to households in the secondary analysis, there was no substantial attenuation in the variance estimates when compared to those from the primary analysis in any of the adjusted models. However, the five-level adjusted variance estimates for underweight and low birthweight (< 2500 g) were higher in the secondary analysis than in the primary analysis, possibly due to the reduced number of households in the sample.

There are four data limitations with this study. First, while NFHS data are considered extremely high quality and representative [31], it should be noted that questions about the covariates included in this study were self-reported and not verified by enumerators, a potential source of measurement error. Child anthropometric data, however, were directly measured and collected by trained field staff, and birthweight was collected using birth records. Second, there were missing observations for the outcome measures and the covariates that were included in the study. These missing observations were excluded from the study, potentially biasing the variance estimates given that the missingness might not have occurred randomly. Third, these are secondary data that were not explicitly collected for the purposes of this study. Fourth, only 5% of the households in our sample had more than one mother. As such, we were unable to disentangle the proportion of variation attributable to households versus mothers within a household. However, supplementary Table 2 shows that for HAZ, the variance estimates are very similar between 5-level and 6-level models, and 6-level models with only households with more than one mother.

Our findings are policy-relevant for several reasons. Decentralization initiated in the early 1990s has expanded the role of states and districts in designing and implementing policies. As an example, the National Nutrition Strategy, implemented by the National Institution for Transforming India, implements district-level interventions as a means to improving child nutrition outcomes [16]. Yet, our findings show that with the exception of HAZ and stunting, a greater proportion of variation in child malnutrition is attributable to states and communities than districts. This is confirmed by our fully adjusted four and five-level models. These findings are consistent with findings from prior studies that also show the relative importance of states and communities compared to districts when examining between-population differences in child malnutrition [11, 13, 32].

Moreover, findings from this paper underscore the importance of households as an important source of clustering and variation in child malnutrition outcomes. This has also been found previously [33], albeit in a different context, and is exemplified by the fact that of the four levels children are nested in, households accounted for the largest source of variation for all outcomes in both the unadjusted and adjusted models. This is further reflected by the fact that when the household level is omitted in the four-level models, the variance estimates at the community and child levels are overestimated by 11 to 50% and 16 to 26%, respectively. The implications of this finding are that between-population differences, such as community, district, or state level contextual factors, are still important determinants of child health. Yet the environments within those geographic levels that people are embedded in, such as households, also function as sources of variation in child malnutrition. This is a particularly important implication given the heterogeneity of child malnutrition outcomes within geographic levels [32]. Therefore, our findings signal the importance of designing policies and interventions that account for the most vulnerable individuals and households within a population in addition to addressing contextual factors at the state and community levels aimed at lowering mean rates of child malnutrition between populations [17–20]. Doing so could help alleviate between and within population disparities in child malnutrition throughout India.

That our findings highlight households as a significant source of variation in child malnutrition outcomes should not undermine efforts aimed at addressing the upstream and social determinants of child malnutrition. This is an important point given that the utility of multilevel modeling is to draw attention away from individuals and households in order to examine place-based effects on health [26–28]. However, underscoring households as important sites of clustering is not at odds with a social epidemiological approach to improving child malnutrition outcomes. This is highlighted by a growing body of literature which demonstrates the relative importance of upstream factors within households when compared to individual-level factors in determining child malnutrition. For example, recent studies show the relative importance of household socioeconomic status and maternal characteristics over nutrition-specific risk factors, such as child feeding practices [5, 24, 25, 34, 35]. Previous work also suggests that investing in early nutrition for girls at the household level is important given that maternal height is strongly associated with child height in the subsequent generation [7, 23, 36]. To this end, a recent study found that children born to mothers who received consistent meals in school had significantly higher HAZ scores than children born to mothers who did not receive this benefit [37]. Another recent study from Odisha shows that providing conditional cash transfers to mothers in poor households can also lead to significant reductions in stunting and anemia among children under five [38]. Evidence suggests that cash transfers can empower women who then invest in the health and wellbeing of their children [8, 24, 39]. Future research should examine the associations between other housing environmental conditions, such as sanitation, clean cooking fuel, and refrigeration, all of which are associated with faltering child growth outcomes [40–42].

## Conclusion

The purpose of this paper was to examine the proportion of variation in child stunting, wasting, underweight, anemia, and low birthweight attributable to the household level relative to commonly analyzed levels such as communities, districts, and states. Overall, we found that of the four levels that children were nested in (households, communities, districts, and states), the greatest proportion of variation in child HAZ, WAZ, WHZ, hemoglobin, birthweight, stunting, underweight, wasting, anemia, and low birthweight was attributable to households. Furthermore, we found that when the household level is omitted from models, the variance estimates for communities and children are overestimated. This implies that households are an important lower-level responsible for a considerable proportion of the variation in child malnutrition.

## Supplementary Information

**Additional file 1: Supplementary Table 1**: Prevalence of anemia among children in households with more than one child.

**Additional file 2: Supplementary Table 2**: Comparison of variance estimates for HAZ between 5-level and 6-level models, and 6-level models with only households with more than one mother.

## Data Availability

The data that support the findings of this study are openly available in India: Standard DHS, 2015–2016 Dataset at https://dhsprogram.com/data/dataset/India_Standard-DHS_2015.cfm?flag=0.
