# Supplementary material for: The relative importance of households as a source of variation in child malnutrition: a multilevel analysis in India
Source: Int J Equity Health. 2021 Oct 12;20:225. doi: 10.1186/s12939-021-01563-7 (PMC8507104; doi:10.1186/s12939-021-01563-7)
Supplement: Supplementary file 1 [file 12939_2021_1563_MOESM1_ESM.docx]

**Total number of households with complete hemoglobin data**	157,734
**Number of households from this sample that have more than one child**	45,043
**Number of households with more than one child with all children anemic**	16,745 (37%)
**Number of households with more than one child with no children anemic**	9,471 (21%)
