# Supplementary material for: The relative importance of households as a source of variation in child malnutrition: a multilevel analysis in India
Source: Int J Equity Health. 2021 Oct 12;20:225. doi: 10.1186/s12939-021-01563-7 (PMC8507104; doi:10.1186/s12939-021-01563-7)
Supplement: Supplementary file 2 [file 12939_2021_1563_MOESM2_ESM.docx]

	5 Level Adjusted HAZ	6 Level adjusted HAZ	N	6 Level Adjusted HAZ only HH with >1 mothers	N
**State**	0.02 (0.007)	0.02 (0.01)	36	0.02 (0.01)	36
**District**	0.03 (0.003)	0.03 (0.003)	640	0.02 (0.01)	636
**Community**	0.15 (0.005)	0.15 (0.004)	27980	0.06 (0.04)	6671
**Household**	0.41 (0.01)	0.41 (0.01)	150279	0.42 (0.05)	8235
**Mothers**	-	0.002 (0)	157917	0.003 (0.001)	15873
**Child**	1.93 (0.01)	1.93 (0.01)	204979	1.94 (0.03)	20302
